# Kaempferol-Mediated Sensitization Enhances Chemotherapeutic Efficacy of Sorafenib Against Hepatocellular Carcinoma: An *In Silico* and *In Vitro* Approach

**DOI:** 10.34172/apb.2020.058

**Published:** 2020-05-11

**Authors:** Bhagyalakshmi Nair, Ruby John Anto, Sabitha M, Lekshmi R. Nath

**Affiliations:** ^1^Amrita School of Pharmacy, Amrita Vishwa Vidyapeetham, AIMS Health Science Campus, Ponekkara P.O., Kochi, Kerala 682041, India.; ^2^Division of Cancer Research, Rajiv Gandhi Center for Biotechnology, Thycaud, Thiruvananthapuram, Kerala- 695014, India. Introduction

**Keywords:** Hepatocellular carcinoma, Sorafenib, Kaempferol, Multi-drug resistance, Combination therapy, P-gp inhibitors

## Abstract

***Purpose:*** Sorafenib is the sole FDA approved drug conventionally used for the treatment of advanced hepatocellular carcinoma (HCC). Despite of the beneficial use of sorafenib in the treatment of HCC, multidrug resistance still remains a challenge. HCC is inherently known as chemotherapy resistant tumor due to P-glycoprotein (P-gp)-mediated multidrug resistance.

***Methods:*** We studied the interaction energy of kaempferol with human multidrug resistance protein-1 (RCSB PDB ID: 2CBZ) using *in silico* method with the help of BIOVIA Discovery Studio. HepG2 and N1S1 liver cancer cell lines were treated in suitable cell culture media to evaluate the efficacy of kaempferol in chemo-sensitizing liver cancer cells towards the effect of sorafenib. Cell viability study was performed by MTT assay.

***Results:*** *In silico* analysis of kaempferol showed best docking score of 23.14 with Human Multi Drug Resistant Protein-1 (RCSB PDB ID: 2CBZ) compared with positive control verapamil. In*in-vitro* condition, combination of sub-toxic concentrations of both kaempferol and sorafenib produced 50% cytotoxicity with concentration of 2.5 µM each which indicates that kaempferol has the ability to reverse the MDR by decreasing the over-expression of P-gp.

***Conclusion:*** Kaempferol is able to sensitize the HepG2 and N1S1 against the sub-toxic concentration of sorafenib. Hence, we consider that the efficacy of sorafenib chemotherapy can be enhanced by the significant approach of combining the sub-toxic concentrations of sorafenib with kaempferol. Thus, kaempferol can be used as a better candidate molecule along with sorafenib for enhancing its efficacy, if validated through preclinical studies.

## Introduction

Hepatocellular carcinoma (HCC) is one of the extreme forms of malignancy characterized by rapid tumour development and is often detected in the last stage where tumour becomes very advanced.^1-3^ Several anti-cancer agents fail to show its efficacy due to multidrug resistance developed by cancerous cells.^4^ HCCs are highly resistant to chemotherapy due to the over-expression of P-glycoprotein (P-gp).^5,6^ P-gp, a unique ATP dependent membrane transport protein is functionally associated with the efflux mechanism of xenobiotics.^7-9^ Over-expression of P-gp lessens the efficacy of several chemotherapeutic agents by depreciating the intracellular concentration of drugs.^7,8^ Thus, the modulation of P-gp can improve the therapeutic efficacy of several anti-neoplastic agents by increasing their retention time inside the tumour cells.^10,11^ Studies reveal that there are several naturally occurring compounds capable of inhibiting the over-expression of P-gp.^11,12^ Flavonoids are class of poly-phenolic herbal compounds that has the potential to inhibit the P-gp.^6,11^ The planar structure and hydrophobic nature of flavonoids are considered as the paramount factors responsible for the interaction with P-gp.^12^ Kaempferol, a dietary flavonoid is well known for its efficacy in inhibiting the P-gp.^13-15^ *In silico* study of the above compound was performed with human multidrug resistant protein-1.^16^ Kaempferol was found to have good interaction energy with a docking score of 23.14. From the *in-vitro* studies, we found that 2.5 µM is a sub -toxic concentration of kaempferol and sorafenib and the aforementioned individual concentration was found to have cell viability of 75%-80% and 65%-70% in N1S1 murine liver cancer cell line and HepG2 liver cancer cell line respectively. In this study, we summarize that the combination is better than sorafenib monotherapy.

## Materials and Methods

### Protein preparation

The crystal structure of human multidrug resistant (MDR) protein 1 was obtained from Protein Data Bank (RCSB PDB ID: 2CBZ) with well-defined whole crystallized fragment of residues 642–871 and resolution of 3-1.5A⁰.^16^ Water molecules were removed from the crystal structure. The pH was identified from the protein report and the protein was prepared by using Biovia discovery studio. The prepared protein was then used for the active site identification.^16^

### Ligand preparation

The ligand molecules kaempferol (PubChem CID: 5280863) and Verapamil (PubChem CID: 2520)^12,17^ were downloaded from PubChem database in the three-dimensional confirmation.

### ADME prediction

ADME properties of the ligand molecule were calculated by using ADMET properties option in Biovia discovery studio.

### Cell lines

The human liver cancer cell line, HepG2 was obtained from NCCS, Pune and the murine liver cancer cell line, N1S1 was a kind gift from Dr. Manzoor K, Centre for Nanoscience, Amrita Institute of Medical Sciences, Kochi, Kerala, India.

### Chemicals

Dulbecco’s Modified Eagle’s medium from Thermo Fisher Scientific (Gibco, NY, USA), 10% fetal bovine serum (Gibco, NY, USA) and 1X antibiotic-antimycotic solution of composition 10 000 units/mL of penicillin, 10 000 µg/mL of streptomycin, and 25 µg/mL of Gibco Amphotericin B from Thermo Fisher Scientific (Gibco, NY, USA), kaempferol was purchased from Sigma Chemicals (St. Louis, MO, USA). Sorafenib was purchased from Santacruz Biotechnology (Santa Cruz, CA, USA), Inc.

### Maintenance of hepatoma cell lines for the experiment

The HepG2 liver cancer cell line and N1S1 murine liver cancer cell line was maintained using Dulbecco’s Modified Eagle’s medium supplemented with fetal bovine serum (FBS) and 1X antibiotic-antimycotic. The cells were maintained at 37°C with 5% CO_2_ in a humidified atmosphere. To maintain sub-confluent state, the cells were sub-cultured twice in a week using 0.1% trypsin with 0.5 mMol EDTA.

### Cell viability assay

3-(4, 5-dimethylthiazol-2-yl)-2, 5-diphenyltetrazolium bromide (MTT) colorimetric assay was used to screen the activity of sorafenib as monotherapy and in combination with kaempferol on two different primary malignant liver cancer cells. Briefly, the cells were seeded into 96-well well plates. After 24 hours of incubation both the cell line was subjected to different concentration of sorafenib (1.25, 2.5, 5, 10 µM) and kaempferol (6.25, 12.5, 25, 50 µM) respectively. The cells were further incubated for 72 hours. Following washing with phosphate buffer saline (PBS), the cells were incubated with MTT solution (100 μL each well) for 2 hours. After 2 hours of incubation, blue coloured crystals were observed which indicates the formation of formazan salts. MTT (3-(4,5-dimethylthiazol-2-yl)-2,5-diphenyltetrazolium bromide) was removed, followed by addition of 100μl solubilising buffer (composition: 5 mL Triton-X 100, 45mL Isopropanol, 12 M HCl (37%). The absorbance was measured after 1hr using microplate reader (Wallac 1420 Multilabel counter, PerkinElmer) at a wavelength of 570 nm.^18,19^

## Results and Discussion

### In silico analysis of flavonoids based on planar ring system with human MDR protein-1 having ATPase binding site

**Figure 1:**
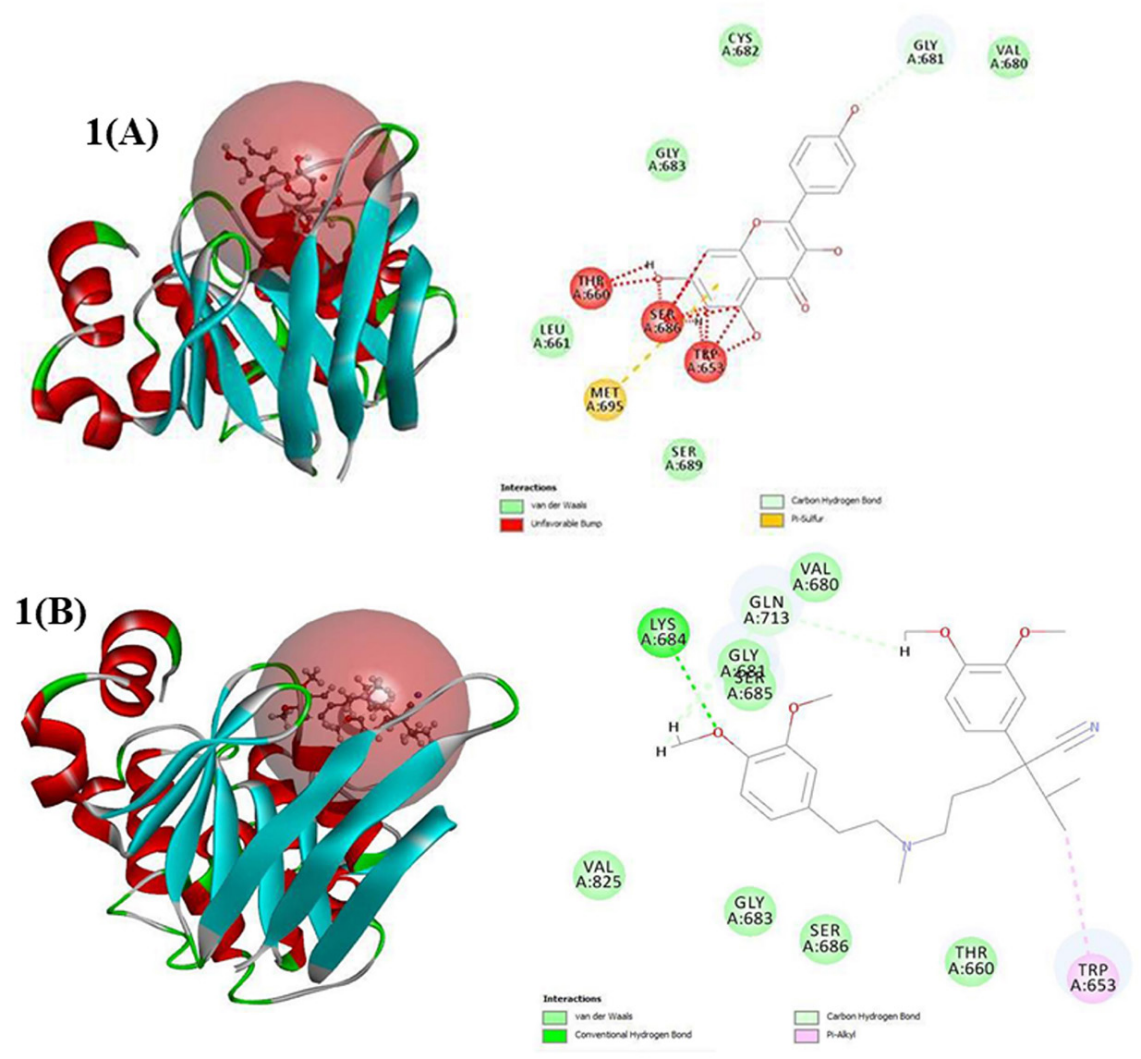
Effect of interaction of different amino acid residues of Human Multidrug Resistant Protein- 1 (RCSB PDB ID: 2CBZ) with kaempferol and verapamil, standard P-glycoprotein inhibitor. (**A)**The docking energy as well as the interaction energy of kaempferol was found to be 23.14 and 27.46 respectively. The light green color indicates the carbon hydrogen bond of interaction between glycine amino acid sequences and the oxygen. The interaction of kaempferol with different amino acid sequences were found to be more when compared with other selected flavonoid compounds(data not shown). (**B)**The docking energy as well as the interaction energy of verapamil was found to be 6.69 and 34.82 respectively. The docking energy of verapamil is comparatively less than kaempferol. It shows van der Waals force of interaction, carbon hydrogen bond interaction etc. with different amino acid sequences of human multidrug resistant protein-1.

Computational studies are nowadays considered as an emerging tool in the drug development process. Nowadays, computational studies are scrutinized as an emerging tool in drug development process. Here, *in silico* analysis was performed using BIOVIA Discovery Studio. The human multidrug resistant (MDR) protein-1 was selected from protein data bank.^16^ The protein was prepared with the help of BIOVIA Discovery Studio and the active site was identified. Flavonoids are a class of compounds having wide range of activities. The presence of planar ring system and the hydrophobic nature of flavonoids are considered as distinctive features in the P-gp inhibition.^12^ Among the putative flavonoid compounds, kaempferol was found to have good interaction energy with the selected Human MDR protein-1 with a docking score of 23.14. From the *in silico* analysis we presume that kaempferol can be used as a better candidate molecule for increasing the therapeutic efficacy of sorafenib (Figure 1A and 1B).

### Combination studies of kaempferol and sorafenib in N1S1, murine hepatoma cell line as well as in HepG2, human hepatoma cell line

**Figure 2:**
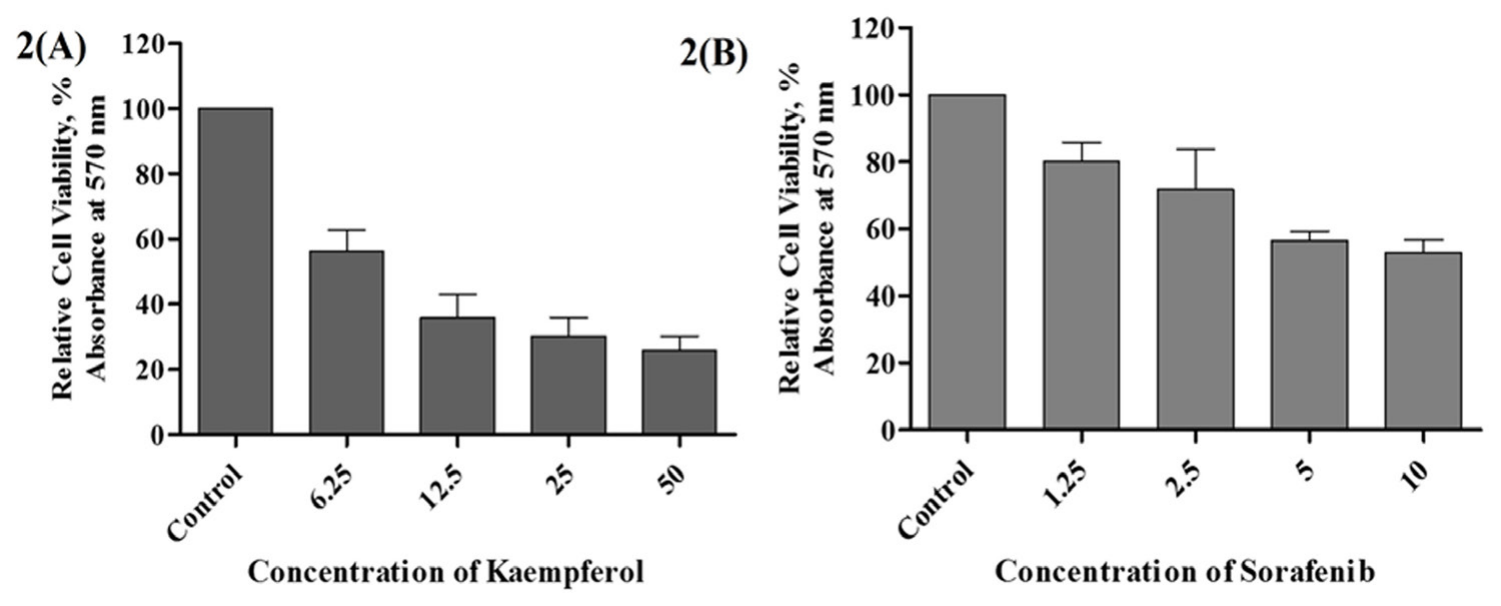
**Dose dependent effect of kaempferol and sorafenib in liver cancer cells, HepG2.** HepG2 cells were treated with kaempferol and sorafenib individually for 72 h and cell viability was assessed by MTT assay as described under “Experimental Procedures.” (A) Kaempferol induced cytotoxicity with an IC50 9.61µM in HepG2 cells. (**B)** sorafenib induced a dose dependent cytotoxicity on HepG2 cells.

**Figure 3:**
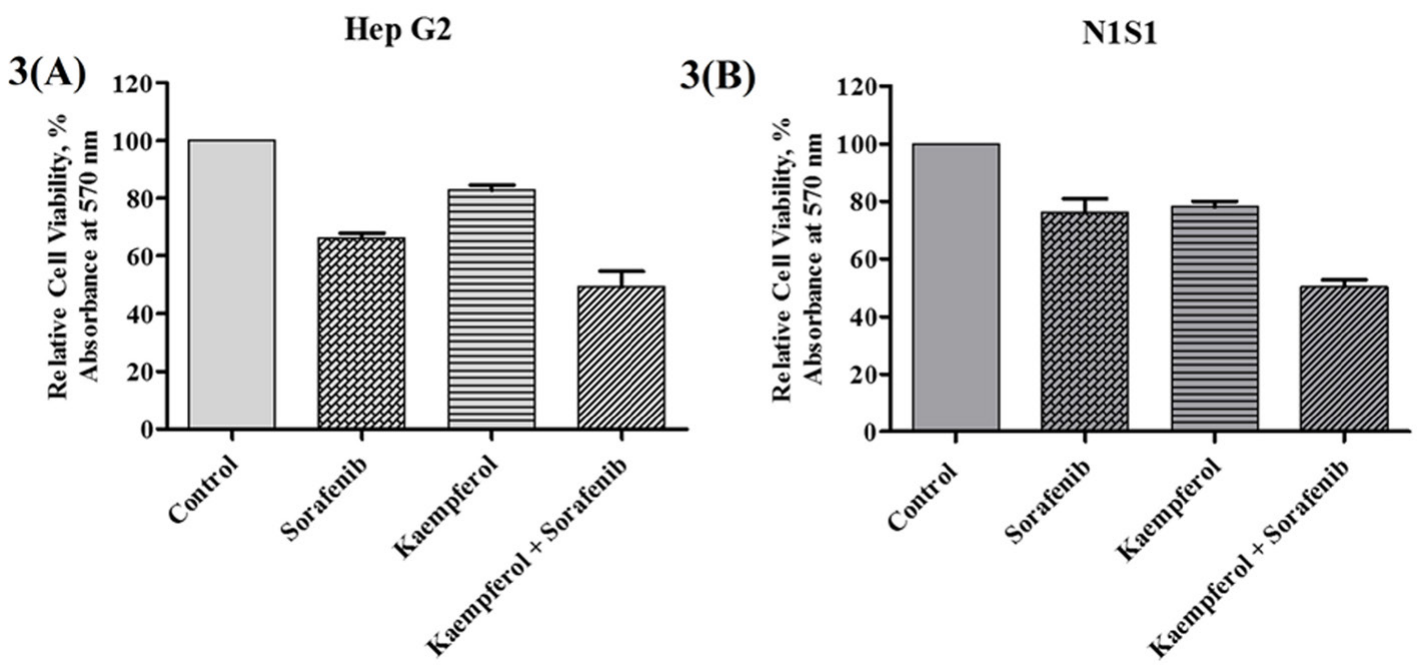
**Combinatorial effect of kaempferol with sorafenib on cell viability in human liver cancer cells, HepG2 and murine liver cancer cells, N1S1:** HepG2 cells were treated with kaempferol and sorafenib individually for 72 h and cell viability was assessed by MTT assay as described under “Experimental Procedures.” **(A)**In HepG2, a combination of 2.5µM sorafenib and 2.5µM kaempferol showed 50% inhibition of cancerous cells with co-incubation of both. **(B**) In N1S1, a combination of 2.5µM sorafenib and 2.5µM kaempferol showed 50% inhibition of cancerous cells with co-incubation of both.

Sorafenib is known to be the first line agent used for HCC but has severe toxicity. Therefore, the therapeutic potential of sorafenib should be improved using the compound at lower doses in combination with naturally occurring phytochemicals. Cell viability studies were conducted to evaluate the sub-toxic concentrations of kaempferol and sorafenib (Figure 2A and 2B). Different set of combinations were evaluated to study the effect of the phytochemical in sensitizing liver cancer cells. The sub-optimal concentration was found to be 2.5 µM for sorafenib with cytotoxicity approx 23.83% (N1S1) and 34.78% (HepG2) after 72 hours treatment. In murine cell line N1S1, a combination of 2.5μM sorafenib and 2.5μM kaempferol showed an approximately 50% inhibition of cancerous cells in two different mode of sensitization such as pre-treatment (data not shown) and co incubation (Figure 3B) respectively. In HepG2 cells, combination of sorafenib with kaempferol produced 67.5% (pre-treatment mode) (data not shown) and 50.43% (co-incubation mode) (Figure 3A) of cell viability at 72 hours.

## Conclusion

Increased failure of chemotherapy in HCC is due to the intrinsic and acquired multi drug resistance. There are several anti-cancer agents, which fail to show its action due to multi drug resistance. In such circumstances, there are increased chances of developing toxicity. HCC is the third most frequent cause of deaths worldwide. Its incidence rates are higher in men than in women, and have doubled in both sexes over the past two decades. Sorafenib possess several side effects, so we aim to reduce the dose of sorafenib by combining it with naturally occurring phytochemicals at non-toxic concentrations. In the present study, we have evaluated a combination of sorafenib and kaempferol using *in silico* as well as *in vitro* studies where the *in-silico* analysis, which suggest that kaempferol is a potent compound that can be used in combination with sorafenib. In computational studies, it is revealed that kaempferol is the only molecule, which has interaction energies among the five selected compounds and has docking score much greater than the positive control, Verapamil, a potent P-gp inhibitor. By analysing the *in vitro* studies, we first standardized the sub-toxic concentration of kaempferol and sorafenib in the *in vitro* culture system and then further focused on choosing the combination strategy. We screened the compounds individually and in combination in two hepatoma cell lines, human-derived HepG2 and murine derived N1S1, to evaluate the efficacy of the combination by MTT assay, which is a very convenient method for assessing drug sensitivity. The combination of kaempferol (2.5μM) and sorafenib (2.5μM) induced an approx. 50% cytotoxic effect in both N1S1 and HepG2 cell lines at 72 hours. The study showed that combination of sorafenib with kaempferol is most effective than sorafenib monotherapy if validated further with detailed *in vivo* studies. The present study indicates that kaempferol may be evaluated as an effective chemo-sensitizer along with sorafenib chemotherapy for HCC.

## Ethical Issues

Not applicable.

## Conflict of Interest

The authors have declared no conflict of interests.

## Acknowledgments

We acknowledge the support from Amrita Vishwa Vidyapeetham for SEED Start up Grant (Project ID: K-PHAR-18-576) to LRN. We thank Dr Shanthi Kumar. V. Nair, Dean of Research, AIMS for the facilities provided. We thank Dr Ram Mohan P.R and Dr Haritha H Nair for proof reading of the manuscript. Ms. Ragima Natesh, Centre for Nanoscience, AIMS for the timely help.
